# Collective states in a ring network of theta neurons

**DOI:** 10.1098/rspa.2021.0817

**Published:** 2022-03

**Authors:** Oleh Omel’chenko, Carlo R. Laing

**Affiliations:** ^1^ University of Potsdam, Institute of Physics and Astronomy, Karl-Liebknecht-Str. 24/25, Potsdam 14476, Germany; ^2^ School of Natural and Computational Sciences, Massey University, Private Bag 102-904 NSMC, Auckland, New Zealand

**Keywords:** theta neurons, neural networks, bumps

## Abstract

We consider a ring network of theta neurons with non-local homogeneous coupling. We analyse the corresponding continuum evolution equation, analytically describing all possible steady states and their stability. By considering a number of different parameter sets, we determine the typical bifurcation scenarios of the network, and put on a rigorous footing some previously observed numerical results.

## 1. Introduction

The collective or emergent behaviour of large networks of neurons is a topic of ongoing interest [1,2], with applications to the study of epilepsy [3,4], binocular rivalry [5], visual hallucinations [6,7] and working memory [8–10], among others. One type of network often considered is a ring, where neurons can be thought of as being arranged on a closed curve. This is natural if some property of a neuron is correlated with an angular variable such as heading in a head directional network [11], the direction in the plane to a visual stimulus that is to be remembered [12] or the orientation of a neuron’s receptive field [13].

A variety of model neurons have been considered when studying ring networks including leaky integrate-and-fire [9,10], quadratic integrate-and-fire (QIF) [14–17], more realistic conductance-based models [5,18,19] and theta neurons [20,21]. Note that under a simple transformation, the quadratic integrate-and-fire neuron with infinite threshold and reset is exactly equivalent to a theta neuron. The theta neuron is the normal form for a saddle-node-on-an-invariant-circle (SNIC) bifurcation [22,23], and we will consider networks of theta neurons because they have remarkable mathematical properties, which allow us to perform many calculations exactly. Our results should be broadly applicable to ring networks of other Type-I neurons, i.e. neurons that start firing through a SNIC bifurcation.

Synaptic coupling between neurons is typically of longer range than just nearest-neighbour, so ring networks of neurons often have non-local coupling. This coupling is normally homogeneous, i.e. the strength of connections between two points on the ring depends on only the distance between these points [6].

There are several types of solutions of interest for a ring network of neurons. One is a ‘bump’ state, in which a spatially localized group of neurons is active, with all other neurons in the domain quiescent [10,24,25]. Because of the homogeneous coupling, such bumps can be positioned anywhere on the domain, and thus their position encodes a single angular variable. Such networks are often bistable, with the spatially uniform ‘all-off’ state being another attractor. It is this bistability that is of interest when modelling working memory [8–10,26].

In this paper, we consider a system of N synaptically coupled theta neurons as presented in [20]

1.1 $$\frac{d\theta_{j}}{dt}=1-\cos\theta_{j}+(1+\cos\theta_{j})(\eta_{j}+\kappa I_{j}),\;j=1,\ldots ,N.$$

The excitability parameters $\eta_{j}$ are chosen from a Lorentzian distribution with mean $\eta_{0}$ and width γ≥0

$$g(\eta )=\frac{\gamma }{\pi }\frac{1}{{\left(\eta -\eta_{0}\right)}^{2}+\gamma^{2}}.$$

Note that in the limit γ→0 the distribution g(η) becomes a delta-distribution, therefore in this case we obtain system (1.1) for identical neurons. Each neuron receives a current input

$$I_{j}(t)=\frac{2\pi }{N}\sum_{k=1}^{N}K_{jk}P_{n}(\theta_{k}(t)),$$

which is obtained as a weighted sum of pulses

$$P_{n}(\theta )=a_{n}{\left(1-\cos\theta \right)}^{n}.$$

The positive integer n controls the width of the pulse and the constant

$$a_{n}=\frac{n!}{(2n-1)!!}=\frac{1\cdot 2\cdot 3\cdot \ldots \cdot n}{1\cdot 3\cdot 5\cdot \ldots \cdot (2n-1)}$$

is chosen according to the normalization condition

$$\int_{0}^{2\pi }P_{n}(\theta )\;d\theta =2\pi .$$

The pulse function $P_{n}(\theta )$ models the output pulse created when a neuron fires, i.e. θ increases through π. Note that it is possible to take the limit n→∞, giving delta function coupling [27]. We only consider the case n=2 below, and based on our experience and the work of others [27] we expect that varying n will only move bifurcations in parameter space, not introduce new dynamics. The parameter κ is the overall coupling strength and the weights $K_{jk}$ are defined according to the rule

$$K_{jk}=K(2\pi (j-k)/N)$$

with some even non-constant 2π-periodic function K(x). Following [20], below we use a cosine coupling kernel

1.2 $$K(x)=\frac{1}{2\pi }(1+A\cos x)$$

with A∈R such that A≠0. (Note that for A=0 equations (1.1) describe a population of mean-field (globally) coupled theta neurons as presented in [23].) For A>1, this form of connectivity is often referred to as ‘Mexican hat’, being positive for small x and negative for larger x, while for A<−1 it is of inverted Mexican hat type, being negative for small x and positive for large x. While neurons are either excitatory or inhibitory, connectivity functions of mixed sign are often used to mimic the combined effects of excitatory and inhibitory populations [25].

From an application point of view, for every solution of equation (1.1) it is important to clarify which neurons are quiescent and which are firing. Mathematically, this question is equivalent to calculating the firing rate for neuron k

**Figure 1.**
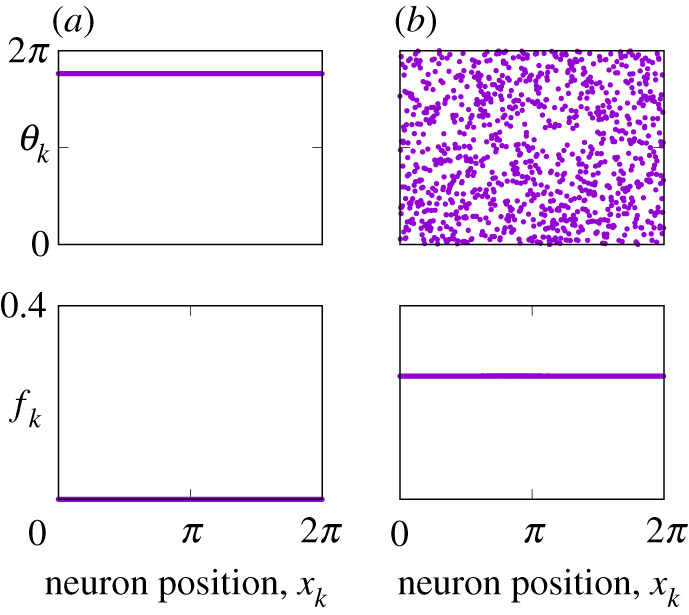
(*a*) Uniform rest state and (*b*) uniform spiking state in equation (1.1). The top panels show snapshots of $\theta_{k}$, the bottom panels show firing rates $f_{k}$. Parameters: N=1024, γ=0, n=2, κ=1, A=3 and $\eta_{0}=-0.2$ (for both states). (Online version in colour.)

1.3 $$f_{k}=\frac{1}{2\pi }{\left\langle \frac{d\theta_{k}}{dt}\right\rangle }_{T},$$

where ${\left\langle \cdot \right\rangle }_{T}$ denotes the time average. For identical neurons (i.e. γ=0) if all $f_{k}$ equal zero, then the corresponding solution of equation (1.1) is called the *uniform rest state*, see figure 1*a*. By contrast, if all $f_{k}$ are equal but positive, then the corresponding solution of equation (1.1) is called the *uniform spiking state*, see figure 1*b*. Note that the coincidence of all firing rates $f_{k}$ does not imply automatically the correlation of the spiking events in neighbouring neurons. In fact, the spikings can occur randomly or following a certain order such that the identical values of $f_{k}$ are obtained only after averaging over time.

**Figure 2.**
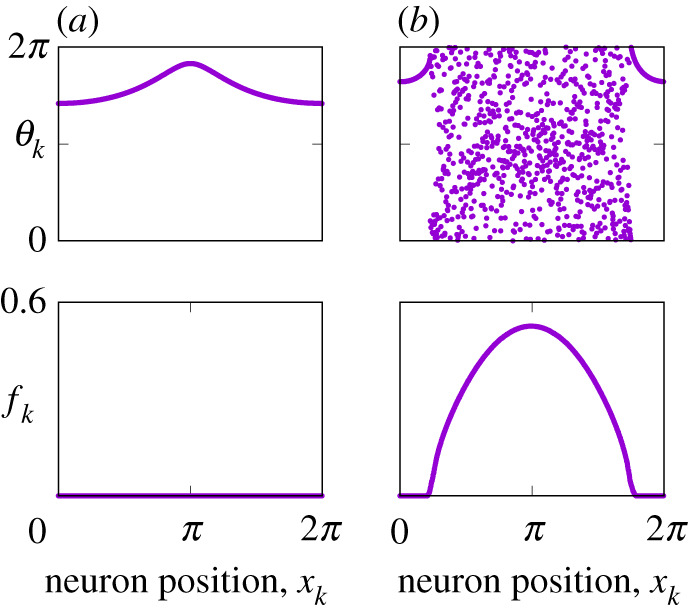
(*a*) Modulated rest state for A=3 and $\eta_{0}=-0.33$, and (*b*) bump state for A=−5 and $\eta_{0}=2$ in equation (1.1). The top panels show snapshots of $\theta_{k}$, the bottom panels show firing rates $f_{k}$. Other parameters: N=1024, γ=0, n=2 and κ=−1. (Online version in colour.)

**Figure 3.**
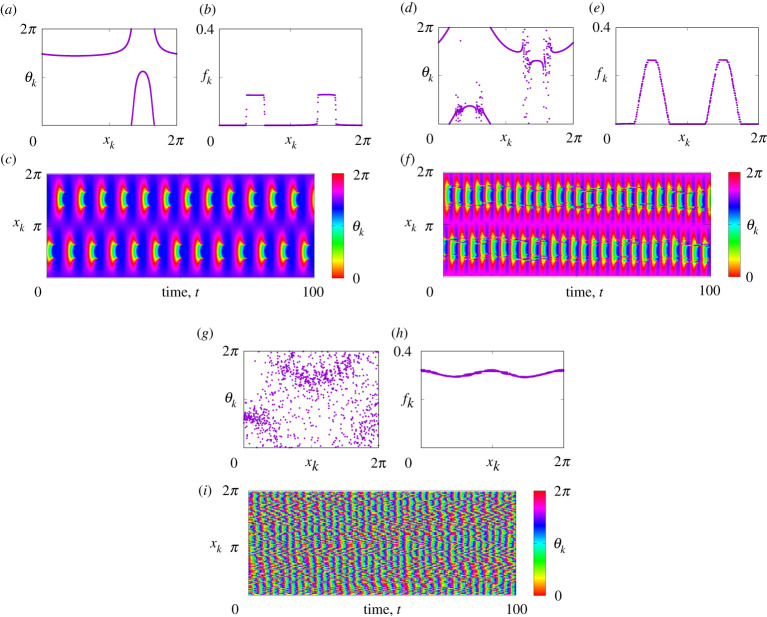
Non-stationary states in equation (1.1) for $\eta_{0}=-2$ (*a*–*c*), for $\eta_{0}=-0.9$ (*d*–*f*) and for $\eta_{0}=-0.3$ (*g*–*i*). The panels (*a*), (*d*), (*g*) show snapshots of $\theta_{k}$, the panels (*b*), (*e*), (*h*) show firing rates $f_{k}$ averaged over 100 time units, and the panels (*c*), (*f*), (*i*) show space–time plots of $\theta_{k}$. Other parameters: N=1024, γ=0, n=2, κ=1 and A=−5.

System (1.1) can also support spatially heterogeneous states, including *modulated rest states*, see figure 2*a*, and *bump states*, see figure 2*b*. Moreover, in system (1.1), one can also observe more complex non-stationary patterns, see figure 3, where more than one group of neurons may be active and their spiking events are modulated not only in space but also in time. Note that the patterns presented in figure 3 occur for parameter values for which all simpler collective states in system (1.1) are unstable. While interesting in their own right, in this paper, we study the existence and stability of only stationary patterns. The structure of the rest of the paper is as follows. In §2 we present the equations governing the continuum limit of (1.1), in §3 we discuss invariant sets of the continuum equations and in §4 we derive self-consistency equations whose solutions describe all stationary states. In §5 we show how to calculate the stability of any steady state and then in §§6 and 7, we use these results to analyse spatially uniform and non-uniform states, respectively. We conclude with a discussion in §8.

## 2. Continuum limit

Using well-established techniques, namely the Ott/Antonsen ansatz [28,29], it was shown in [20] that in the continuum limit (N→∞), the long-term coarse-grained dynamics of system (1.1) can be described by a mean-field equation

2.1 $$\frac{\partial z}{\partial t}=\frac{(i\eta_{0}-\gamma ){\left(1+z\right)}^{2}-i{\left(1-z\right)}^{2}}{2}+\kappa \frac{i{\left(1+z\right)}^{2}}{2}KH_{n}(z),$$

for a local order parameter z(x,t), which represents the value of $e^{i\theta_{j}(t)}$ averaged over all indices j such that 2πj/N≈x∈[0,2π]. Note that the symbol K in equation (2.1) denotes an integral operator

$$(K\varphi )(x)=\int_{0}^{2\pi }K(x-y)\varphi (y)\;dy$$

and

$$H_{n}(z)=a_{n}\left[C_{0}+\sum_{q=1}^{n}C_{q}(z^{q}+{\bar{z}}^{q})\right]$$

is a nonlinear function with

$$C_{q}=\sum_{k=0}^{n}\sum_{m=0}^{k}\frac{\delta_{k-2m,q}{\left(-1\right)}^{k}n!}{2^{k}(n-k)!m!(k-m)!}.$$

While the complex quantity z may not seem to have an obvious biological interpretation, one can use the equivalence of theta and QIF neurons to determine relevant quantities. Defining $W\equiv (1-\bar{z})/(1+\bar{z})$ one can show [21,30] that the instantaneous firing rate of neurons at position x and time t is the flux through θ=π:

2.2 $$f(x,t)=\frac{1}{\pi }\text{Re}\;W=\frac{1-|z{|}^{2}}{\pi |1+z{|}^{2}}.$$

If z(x,t) does not depend on t, then formula (2.2) yields a continuum limit analogue of the averaged firing rates defined by equation (1.3). Similarly, if $V_{j}=\tan\left(\theta_{j}/2\right)$ is the voltage of the jth QIF neuron, the mean voltage at position x and time t is given by Im W.

**Remark 2.1.**

It follows from the above definition that $H_{n}(1)=0$ for every n∈N. Indeed, using the substitution m=k−l, we obtain

$$C_{q}=\sum_{k=0}^{n}\sum_{m=0}^{k}\frac{\delta_{k-2m,q}{\left(-1\right)}^{k}\;n!}{2^{k}(n-k)!m!(k-m)!}=\sum_{k=0}^{n}\sum_{l=0}^{k}\frac{\delta_{-k+2l,q}{\left(-1\right)}^{k}\;n!}{2^{k}(n-k)!(k-l)!l!}=C_{-q}.$$

Therefore,

$$\frac{H_{n}(1)}{a_{n}}=\sum_{q=-n}^{n}C_{q}=\sum_{q=-n}^{n}\sum_{k=0}^{n}\sum_{m=0}^{k}\frac{\delta_{k-2m,q}{\left(-1\right)}^{k}}{2^{k}}\left(\begin{array}{c} n\\ k \end{array}\right)\left(\begin{array}{c} k\\ m \end{array}\right)=0,$$

where we used the identity

$$\sum_{q=-n}^{n}\delta_{k-2m,q}=1\;\text{valid for}\;0\leq m\leq k\leq n$$

and the two other identities

$$\sum_{m=0}^{k}\left(\begin{array}{c} k\\ m \end{array}\right)=2^{k}\;\text{and}\;\sum_{k=0}^{n}{\left(-1\right)}^{k}\left(\begin{array}{c} n\\ k \end{array}\right)=0$$

that follow from the properties of the binomial coefficients.

**Remark 2.2.**

Note that the function $H_{n}(z)$ is real even for a complex argument z. To stress this fact, we can rewrite this function in the form

$$H_{n}(z)=a_{n}C_{0}+2\text{Re}\;D_{n}(z)\;\text{where}\;D_{n}(z)=a_{n}\sum_{q=1}^{n}C_{q}z^{q}.$$

**Remark 2.3.**

Recall that for every continuous kernel K(x) the above operator K is a bounded compact operator on the space of continuous functions C([0,2π];C). Moreover, in the case of the cosine kernel (1.2), for every φ(x)∈C([0,2π];C), we have

(Kφ)(x)=⟨φ⟩+Acos⁡x⟨φcos⁡y⟩+Asin⁡x⟨φsin⁡y⟩,

where

$$\langle \varphi \rangle =\frac{1}{2\pi }\int_{0}^{2\pi }\varphi (y)\;dy.$$

Furthermore, if φ(x) is an even function (i.e. φ(−x)=φ(x)), then

2.3 (Kφ)(x)=⟨φ⟩+Acos⁡x⟨φcos⁡y⟩.

Equation (2.1) can be rewritten in the form

2.4 $$\frac{\partial z}{\partial t}=\frac{(iJ(x,t)-\gamma ){\left(1+z\right)}^{2}-i{\left(1-z\right)}^{2}}{2},$$

where $J(x,t)=\eta_{0}+\kappa KH_{n}(z(x,t))$. Then, for every point $x_{0}\in [0,2\pi ]$ it can be considered as an ODE with respect to $z(x_{0},t)$ with a driving force $J(x_{0},t)$. This fact and the special structure of equation (2.4) have important consequences for the dynamics of solutions to equation (2.1), which we discuss in the next section.

## 3. Invariant sets of equation (2.1)

Let us consider an ordinary differential equation

3.1 $$\frac{du}{dt}=\frac{(iJ(t)-\gamma ){\left(1+u\right)}^{2}-i{\left(1-u\right)}^{2}}{2}$$

with a complex-valued unknown function u(t). In the following, we suppose that J(t) is a real coefficient and γ≥0. Moreover, we denote by D={u∈C:|u|<1} the open unit disc in the complex plane, by $\bar{D}=\{u\in C:|u|\leq 1\}$ the closure of D, and by S={u∈C:|u|=1} the boundary of D.

**Proposition 3.1.**

*The closed unit disc* $\bar{D}$ *is an invariant set of equation* (3.1). *In other words, if* $u(0)\in \bar{D}$, *then the corresponding solution* u(t) *of equation* (3.1) *lies in* $\bar{D}$ *for all* t>0.

*Moreover, if* γ>0, *then the* ω-*limit set of any initial condition* $u(0)\in \bar{D}$ *lies in the open unit disc* D.

*Furthermore, if* γ=0, *then the unit circle* S *and the open unit disc* D *are distinct invariant sets of equation* (3.1).

**Proof.**

The complex conjugate of equation (3.1) reads

$$\frac{d\bar{u}}{dt}=\frac{(-iJ(t)-\gamma ){\left(1+\bar{u}\right)}^{2}+i{\left(1-\bar{u}\right)}^{2}}{2}.$$

Then, simple calculations yield

$$\frac{d|u{|}^{2}}{dt}=u\frac{d\bar{u}}{dt}+\bar{u}\frac{du}{dt}=(J(t)-1)(1-|u{|}^{2})\text{Im}\;u-\gamma (2|u{|}^{2}+(1+|u{|}^{2})\text{Re}\;u).$$

For |u|=1, this equation implies

3.2 $$\frac{d|u{|}^{2}}{dt}=-2\gamma (1+\text{Re}\;u)\leq 0.$$

Hence, if |u(0)|≤1, then |u(t)| cannot grow above one, and therefore |u(t)|≤1 for all t>0.

On the other hand, if γ>0, then for every u∈S except u=−1, the differential inequality (3.2) becomes a strict inequality

$$\frac{d|u{|}^{2}}{dt}<0.$$

Moreover, at u=−1, the right-hand side of equation (3.1) equals −2i, and therefore u=−1 is not an equilibrium of equation (3.1). From all these facts, it follows that any initial condition u(0)∈S is pushed inside the disc D for t>0. Moreover, if u(0)∈D, then u(t) also remains inside D for all t>0.

The last assertion of the proposition follows from a simple observation that

$$\frac{d|u{|}^{2}}{dt}=0,$$

for all |u|=1 and γ=0.

## 4. Self-consistency equation

We will start with an auxiliary proposition.

**Proposition 4.1.**

*For any* c∈R, *the equation*

4.1 $$\frac{du}{dt}=\frac{(ic-\gamma ){\left(1+u\right)}^{2}-i{\left(1-u\right)}^{2}}{2}$$

*has exactly one stable equilibrium in the closed unit disc* $\bar{D}$. *More precisely, if* γ=0, *then the equilibrium is neutrally stable, but if* γ>0 *then it is linearly stable*.

**Proof.**

All equilibria of equation (4.1) satisfy a quadratic equation

$${\left(\frac{1-u}{1+u}\right)}^{2}=c+i\gamma ,$$

which can be rewritten in the equivalent form

4.2 $$\frac{1-u}{1+u}=\xi ,$$

where ξ denotes one of the two distinct values of the square root of c+iγ. The rational function in the left-hand side of equation (4.2) is a particular Möbius transformation, which maps the closed unit circle $\bar{D}$ on to the closed half-plane {u∈C:Re u>0}. Therefore, to get a solution $u\in \bar{D}$ from equation (4.2), we need to choose $\xi =\sqrt{c+i\gamma }$ with Re ξ≥0. In this case, equation (4.2) yields

4.3 $$u=\frac{1-\xi }{1+\xi }.$$

Let us linearize equation (4.1) around the above equilibrium u. In this case, the dynamics of small perturbations v obeys a linear equation

$$\frac{dv}{dt}=[(ic-\gamma )(1+u)+i(1-u)]v.$$

Therefore, the linear stability of equilibrium u requires

Re [(ic−γ)(1+u)+i(1−u)]≤0.

Using (4.2) and (4.3), we convert the expression in square brackets as follows:

$$(ic-\gamma )(1+u)+i(1-u)=i[(c+i\gamma )(1+u)+(1-u)]=i(\xi^{2}+\xi )(1+u)=2i\xi .$$

Therefore, the equilibrium u is (neutrally) stable if and only if Im ξ≥0.

In the appendix, it is shown that for any c∈R and any γ≥0 there exists exactly one value $\xi =\sqrt{c+i\gamma }$ such that Re ξ≥0 and Im ξ≥0. This ends the proof.

Let $U_{\gamma }(c)$ be a function that determines the equilibrium $u\in \bar{D}$ of equation (4.1) mentioned in proposition 4.1. More precisely,

4.4 $$U_{\gamma }(c)=\frac{1-\sqrt{c+i\gamma }}{1+\sqrt{c+i\gamma }},$$

where the square root $\sqrt{c+i\gamma }$ is calculated according to proposition A.1 in the appendix.

**Remark 4.2.**

In the degenerate case γ=0, function $U_{\gamma }(c)$ simplifies as follows:

$$U_{0}(c)=\left\{ \begin{array}{ll} \frac{1-\sqrt{c}}{1+\sqrt{c}} & \text{for}\;c\geq 0,\\ \frac{1-i\sqrt{-c}}{1+i\sqrt{-c}} & \text{for}\;c<0. \end{array}\right.$$

Then, if we calculate the firing rates (2.2) corresponding to $z=U_{0}(c)$, we obtain $f(x,t)=\sqrt{c}/\pi$ for c≥0, and f(x,t)=0 for c<0.

Suppose that a(x) is an equilibrium of equation (2.1). Then a(x) is also an equilibrium of equation (2.4) with $J(x,t)=w(x):=\eta_{0}+\kappa KH_{n}(a)$. Using proposition 4.1, we find $a(x)=U_{\gamma }(w(x))$. Therefore, the last two formulae are consistent with each other iff w(x) satisfies the integral equation

4.5 $$w(x)=\eta_{0}+\kappa KH_{n}(U_{\gamma }(w)).$$

The self-consistency equation (4.5) becomes particularly simple in the case of the cosine kernel K(x). Indeed, if in this case, we look for an even solution of equation (4.5), then due to remark 2.3 we have

4.6 $$w(x)={\hat{w}}_{0}+{\hat{w}}_{1}\cos x,$$

with some coefficients ${\hat{w}}_{0},{\hat{w}}_{1}\in R$. Inserting expressions (2.3) and (4.6) into equation (4.5) and equating the x-independent terms and the terms proportional to cos⁡x separately, we obtain a nonlinear two-dimensional system

4.7 $${\hat{w}}_{0}=\eta_{0}+\kappa \langle H_{n}(U_{\gamma }({\hat{w}}_{0}+{\hat{w}}_{1}\cos y))\rangle$$

and

4.8 $${\hat{w}}_{1}=\kappa A\langle H_{n}(U_{\gamma }({\hat{w}}_{0}+{\hat{w}}_{1}\cos y))\cos y\rangle ,$$

which can be solved approximately with a standard Newton’s method. If we find a pair ${\left({\hat{w}}_{0},{\hat{w}}_{1}\right)}^{\text{T}}\in R^{2}$ that satisfies (4.7) and (4.8), then formula (4.6) yields the corresponding solution of equation (4.5), while the formula $a(x)=U_{\gamma }(w(x))$ yields the corresponding equilibrium of equation (2.1). Note that similar self-consistency equations have been found in similar systems [31–33] but those authors were studying chimera states.

Having seen how to find equilibria of equation (2.1) we now move on to determine their stability.

## 5. Stability of equilibria of equation (2.1)

Suppose that z=a(x) is a stationary solution of equation (2.1). Then, inserting the ansatz z=a(x)+v(x,t) into equation (2.1) and linearizing the resulting relation with respect to small perturbations v(x,t), we obtain a differential equation determining the linear stability of a(x)

5.1 $$\frac{\partial v}{\partial t}=\mu (x)v+\kappa \frac{i{\left(1+a(x)\right)}^{2}}{2}K(D_{n}^{\prime }(a)v+\bar{D_{n}^{\prime }(a)}\bar{v}),$$

where

$$\mu (x)=[i(\eta_{0}+\kappa KH_{n}(a))-\gamma ](1+a(x))+i(1-a(x))$$

and

$$D_{n}^{\prime }(z)=\frac{d}{dz}D_{n}(z)=a_{n}\sum_{q=1}^{n}qC_{q}z^{q-1}.$$

To investigate the decay of different spatial modes, we insert the ansatz

$$v(x,t)=v_{+}(x)\;e^{\lambda t}+{\bar{v}}_{-}(x)\;e^{\bar{\lambda }t}$$

into equation (5.1) and equate separately the terms at $e^{\lambda t}$ and $e^{\bar{\lambda }t}$. Thus, we obtain a spectral problem

5.2 $$\begin{array}{rl} \lambda \left(\begin{array}{c} v_{+}\\ v_{-} \end{array}\right) & \;=\left(\begin{array}{cc} \mu (x) & 0\\ 0 & \bar{\mu (x)} \end{array}\right)\left(\begin{array}{c} v_{+}\\ v_{-} \end{array}\right)+\frac{\kappa i}{2}\left(\begin{array}{cc} {\left(1+a\right)}^{2} & {\left(1+a\right)}^{2}\\ -{\left(1+\bar{a}\right)}^{2} & -{\left(1+\bar{a}\right)}^{2} \end{array}\right)\left(\begin{array}{c} KD_{n}^{\prime }(a)v_{+}\\ K\bar{D_{n}^{\prime }(a)}v_{-} \end{array}\right). \end{array}$$

Note that the right-hand side of equation (5.2) is a sum of two bounded operators on $C([0,2\pi ];C^{2})$. Moreover, the first term of this sum is a multiplication operator, whereas the second term is a compact operator (see remark 2.3). Operators with similar structure have been often considered in the context of studying the chimera states [34]. Using the results of these works, we can draw the following conclusions:

(i) The spectrum determined by equation (5.2) lies in a bounded region of the complex plane.
(ii) The spectrum consists of two parts: essential $\sigma_{\text{ess}}$ and discrete $\sigma_{\text{discr}}$ spectra.
(iii) The essential spectrum $\sigma_{\text{ess}}$ is determined by the multiplication operator in the right-hand side of (5.2). It can be computed explicitly by
  $$\sigma_{\text{ess}}=\{\mu (x):x\in [0,2\pi ]\}\cup \{\text{c.c.}\}.$$
(iv) The discrete spectrum $\sigma_{\text{discr}}$ consists of a finite number of eigenvalues with finite multiplicity.

**Proposition 5.1.**

*Suppose that* $a(x)=U_{\gamma }(w(x))$, *where* w(x) *is a solution of the self-consistency equation* (4.5). *Then the essential spectrum corresponding to* a(x) *is given by*

$$\sigma_{\text{ess}}=\left\{2i\sqrt{w(x)+i\gamma }:x\in [0,2\pi ]\right\}\cup \{\text{c.c.}\}.$$

**Proof.**

Replacing a(x) by $U_{\gamma }(w(x))$ in the definition of μ(x) and using equation (4.5), we obtain

$$\mu (x)=i(w(x)+i\gamma )(1+U_{\gamma }(w(x)))+i(1-U_{\gamma }(w(x))).$$

Then, formula (4.4) yields $\mu (x)=2i\sqrt{w(x)+i\gamma }$.

**Remark 5.2.**

Recall that the branch of the complex square root in (4.4) is chosen so that $\text{Im}\;(\sqrt{w(x)+i\gamma })\geq 0$. Therefore, $\text{Re}\;(2i\sqrt{w(x)+i\gamma })\leq 0$ and hence the essential spectrum $\sigma_{\text{ess}}$ in proposition 5.1 is always neutrally stable. Moreover, for γ>0 the value of $\sqrt{w(x)+i\gamma }$ cannot be real, therefore in this case, the essential spectrum $\sigma_{\text{ess}}$ lies entirely in the open half-plane {λ∈C:Re λ<0}.

The spectral problem (5.2), in general, is infinite-dimensional. However, in the case of cosine kernel (1.2), it can be reduced to a finite-dimensional form. Indeed, remark 2.3 implies that

5.3 $$\left(\begin{array}{c} KD_{n}^{\prime }(a)v_{+}\\ K\bar{D_{n}^{\prime }(a)}v_{-} \end{array}\right)=\sum_{k=1}^{3}V_{k}\phi_{k}(x),$$

where $V_{k}\in C^{2}$, and $\phi_{1}(x)=1$, $\phi_{2}(x)=\cos x$, $\phi_{3}(x)=\sin x$ are three linearly independent functions that span the range of the operator K. Inserting (5.3) into equation (5.2) and expressing ${\left(v_{+},v_{-}\right)}^{\text{T}}$ from the resulting equation, we obtain

5.4 $$\left(\begin{array}{c} D_{n}^{\prime }(a)v_{+}\\ \bar{D_{n}^{\prime }(a)}v_{-} \end{array}\right)=\sum_{k=1}^{3}\text{L}(x,\lambda )V_{k}\phi_{k}(x),$$

where

$$\text{L}(x,\lambda )=\left(\begin{array}{cc} D_{n}^{\prime }(a) & 0\\ 0 & \bar{D_{n}^{\prime }(a)} \end{array}\right){\left(\begin{array}{cc} \lambda -\mu (x) & 0\\ 0 & \lambda -\bar{\mu (x)} \end{array}\right)}^{-1}\frac{\kappa i}{2}\left(\begin{array}{cc} {\left(1+a\right)}^{2} & {\left(1+a\right)}^{2}\\ -{\left(1+\bar{a}\right)}^{2} & -{\left(1+\bar{a}\right)}^{2} \end{array}\right)$$

is a (2×2)-matrix. Now, using remark 2.3 and the definition of functions $\phi_{k}(x)$ given after (5.3), we conclude

$$\left(\begin{array}{c} KD_{n}^{\prime }(a)v_{+}\\ K\bar{D_{n}^{\prime }(a)}v_{-} \end{array}\right)=\sum_{k=1}^{3}\left(\langle \text{L}(x,\lambda )\phi_{1}(x)\phi_{k}(x)\rangle \phi_{1}(x)+\sum_{j=2}^{3}A\langle \text{L}(x,\lambda )\phi_{j}(x)\phi_{k}(x)\rangle \phi_{j}(x)\right)V_{k}.$$

Because of the linear independence of $\phi_{j}(x)$, this formula agrees with (5.3) if and only if

5.5 $$V_{j}=\sum_{k=1}^{3}{\text{B}}_{\text{jk}}(\lambda )V_{k},$$

where

$${\text{B}}_{\text{1k}}(\lambda )=\langle \text{L}(x,\lambda )\phi_{1}(x)\phi_{k}(x)\rangle$$

and

$${\text{B}}_{\text{jk}}(\lambda )=A\langle \text{L}(x,\lambda )\phi_{j}(x)\phi_{k}(x)\rangle \;\text{for}\;j=2,3.$$

(Note that in the above expressions the averaging over x is performed for each matrix element separately.) Finally, we see that system (5.5) has a non-zero solution ${\left(V_{1},V_{2},V_{3}\right)}^{\text{T}}$ if and only if

5.6 $$\det({\text{I}}_{6}-\text{B}(\lambda ))=0,$$

where

$$\text{B}(\lambda )=\left(\begin{array}{ccc} {\text{B}}_{\text{11}}(\lambda ) & {\text{B}}_{\text{12}}(\lambda ) & {\text{B}}_{\text{13}}(\lambda )\\ {\text{B}}_{\text{21}}(\lambda ) & {\text{B}}_{\text{22}}(\lambda ) & {\text{B}}_{\text{23}}(\lambda )\\ {\text{B}}_{\text{31}}(\lambda ) & {\text{B}}_{\text{32}}(\lambda ) & {\text{B}}_{\text{33}}(\lambda ) \end{array}\right)$$

and ${\text{I}}_{6}$ is the (6×6) unit matrix. Equation (5.6) is a characteristic equation that determines the discrete spectrum $\sigma_{\text{discr}}$ associated with any equilibrium of equation (2.1) in the case of cosine coupling kernel (1.2).

In the next section, we consider the existence of spatially uniform steady states of equation (2.1) and use the results in this section to determine their stability.

## 6. Spatially uniform states

### (a) Existence of uniform states

It is easy to verify that for any cosine kernel (1.2) (in fact, even for any kernel that satisfies $\int_{0}^{2\pi }K(x)\;dx=2\pi$) equation (4.5) has a set of spatially uniform solutions

w(x)=p∈R,

where the constant p satisfies $p=\eta_{0}+\kappa H_{n}(U_{\gamma }(p))$, or equivalently

6.1 $$\eta_{0}=p-\kappa H_{n}(U_{\gamma }(p)).$$

By construction, formula (6.1) yields a parametric representation of all uniform states of equation (2.1) with stable essential spectra. Indeed, for every $(p,\kappa )\in R^{2}$, we can find the corresponding excitability parameter $\eta_{0}$ using equation (6.1) and the corresponding equilibrium of equation (2.1) by using the formula $U_{\gamma }(p)$. Importantly, it follows from remark 4.2 that for γ=0 the sign of the parameter p allows us to distinguish between the uniform spiking states (p>0) and the uniform rest states (p<0). Moreover, for small values of γ, the same criterion can be used to distinguish between the partially synchronized spiking states and the partially synchronized rest states introduced in [23].

Formula (6.1) gives a global representation of the branch of uniform states. However, in practice, it is more important to answer the other question: How many different uniform states can be found in equation (2.1) for given parameters $(\eta_{0},\kappa )\in R^{2}$ and what are their types? This question can be answered geometrically, if we rewrite equation (6.1) in the form

6.2 $$\frac{p-\eta_{0}}{\kappa }=F_{n,\gamma }(p),$$

where

$$F_{n,\gamma }(p)=H_{n}(U_{\gamma }(p))=a_{n}C_{0}+2\text{Re}\;(D_{n}(U_{\gamma }(p))).$$

Note that the right-hand side of equation (6.2) does not depend on either $\eta_{0}$ or κ. Moreover, for any fixed γ≥0 we have $U_{\gamma }(p)\to -1$ as p→±∞. This implies that $F_{n,\gamma }(p)\to H_{n}(-1)$ as p→±∞, and therefore for any κ∈R and any $\eta_{0}\in R$, equation (6.2) has at least one real solution. In the next proposition, we show that under certain conditions equation (6.2) can also have three different roots.

**Proposition 6.1.**

*Let the following conditions be satisfied*:

(a) $F_{n,0}^{\prime }(p)\to +\infty$ *for* p→+0,
(b) $F_{n,0}^{\prime }(p)\to 0$ *for* p→+∞,
(c) $F_{n,0}^{\prime \prime }(p)<0$ *for* p>0,
(d) $F_{n,0}^{\prime }(p)\to 0$ *for* p→−0 *and for* p→−∞,
(e) *there exists* $p_{\text{min}}<0$ *such that* $F_{n,0}^{\prime \prime }(p)<0$ *for* $p<p_{\text{min}}$ *and* $F_{n,0}^{\prime \prime }(p)>0$ *for* $p_{\text{min}}<p<0$.

*Then, equation* (6.2) *with* γ=0 *has the following properties*:

(i) *For every* κ>0, *there exists* $p_{0}>0$ *such that* $F_{n,0}^{\prime }(p_{0})=1/\kappa$. *Moreover*,
  — *if* $\eta_{0}>0$, *then equation* (6.2) *has one positive root*,
  — *if* $p_{0}-\kappa F_{n,0}(p_{0})<\eta_{0}<0$, *then equation* (6.2) *has two distinct positive roots and one negative root*,
  — *if* $\eta_{0}<p_{0}-\kappa F_{n,0}(p_{0})$, *then equation* (6.2) *has one negative root*.
(ii) *If* $1/F_{n,0}^{\prime }(p_{\text{min}})<\kappa <0$ *and* $\eta_{0}<0$, *then equation* (6.2) *has one negative root. On the other hand, if* $1/F_{n,0}^{\prime }(p_{\text{min}})<\kappa <0$ *and* $\eta_{0}>0$, *then equation* (6.2) *has one positive root*.
(iii) *For every* $\kappa <1/F_{n,0}^{\prime }(p_{\text{min}}),$ *there exist* $p_{1}<p_{2}<0$ *such that* $F_{n,0}^{\prime }(p_{1,2})=1/\kappa$. *Moreover*,
  — *if* $\eta_{0}<p_{2}-\kappa F_{n,0}(p_{2})$ *or* $p_{1}-\kappa F_{n,0}(p_{1})<\eta_{0}<0$, *then equation* (6.2) *has one negative root*,
  — *if* $0<\eta_{0}<p_{1}-\kappa F_{n,0}(p_{1})$, *then equation* (6.2) *has two distinct negative roots and one positive root*,
  — *if* $p_{2}-\kappa F_{n,0}(p_{2})<\eta_{0}<\min\{0,p_{1}-\kappa F_{n,0}(p_{1})\}$, *then equation* (6.2) *has three distinct negative roots*,
  — *if* $\eta_{0}>\max\{0,p_{1}-\kappa F_{n,0}(p_{1})\}$, *then equation* (6.2) *has one positive root*.

**Proof.**

**Figure 4.**
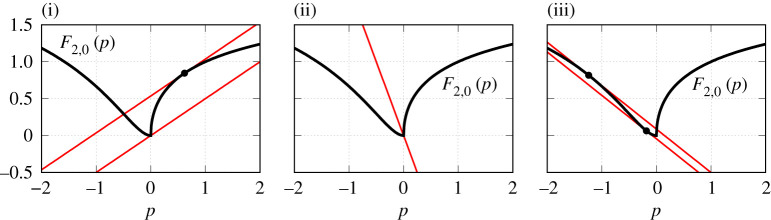
Geometric solution of equation (6.2) with γ=0 and n=2 for several values of the coupling strength κ. The thick black curve shows the graph of $F_{2,0}(p)$. Solid thin red lines show ‘extreme’ lines $(p-\eta_{0})/\kappa$. Panels (i)–(iii) illustrate how the number and sign of the roots of equation (6.2) change for varying parameter κ. (Online version in colour.)

Suppose κ>0. Then the existence of a unique value $p_{0}>0$ such that $F_{n,0}^{\prime }(p_{0})=1/\kappa$ follows from the assumptions (a)–(c). The remaining part of the assertion (i) follows from the geometric consideration of equation (6.2) explained in figure 4. Indeed, if the slope κ is fixed, then changing $\eta_{0}$ we can encounter three qualitatively different situations, which are separated by two critical cases: the line $(p-\eta_{0})/\kappa$ is tangent to the graph of $F_{n,0}(p)$ for p>0, or this line passes through the point (0,0).

Two other assertions (ii) and (iii) are proved by a similar geometric argument.

**Remark 6.2.**

Assumptions (d) and (e) of proposition 6.1 imply that $p_{1}(\kappa )$ and $p_{2}(\kappa )$ are smooth monotonic functions for $\kappa <1/F_{n,0}^{\prime }(p_{\text{min}})$. Moreover,

6.3 $$p_{1}(\kappa )-\kappa F_{n,0}(p_{1}(\kappa ))\geq p_{2}(\kappa )-\kappa F_{n,0}(p_{2}(\kappa )).$$

Indeed, by definition we have $\kappa =1/F_{n,0}^{\prime }(p_{1}(\kappa ))$, therefore the Implicit Function Theorem implies that

$$\frac{dp_{1}}{d\kappa }=-\frac{{\left[F_{n,0}^{\prime }(p_{1}(\kappa ))\right]}^{2}}{F_{n,0}^{\prime \prime }(p_{1}(\kappa ))}.$$

Hence, using the chain rule, we obtain

ddκ(p1(κ)−κFn,0(p1(κ))∑)=ddp(p−Fn,0(p)Fn,0′(p))|p=p1(κ)⋅dp1dκ=−Fn,0(p1(κ)).

A similar formula can be also obtained for $p_{2}(\kappa )$, therefore

ddκ(p1(κ)−κFn,0(p1(κ))−p2(κ)+κFn,0(p2(κ))∑)=Fn,0(p2(κ))−Fn,0(p1(κ))≤0,

where we used the fact that $F_{n,0}(p)$ is a decreasing function for p<0 (see assumptions (d) and (e)). Since the relation (6.3) is satisfied for $\kappa =1/F_{n,0}^{\prime }(p_{\text{min}})$ (in this case $p_{1}(\kappa )=p_{2}(\kappa )$), it obviously will remain true for all smaller values of κ.

**Remark 6.3.**

It is easy to verify that the assumptions of proposition 6.1 are satisfied for n=2. In particular,

$$H_{2}(z)=1+2\text{Re}\;D_{2}(z)\;\mathrm{and}\;D_{2}(z)=-\frac{2}{3}z+\frac{1}{6}z^{2},$$

and

$$F_{2,0}^{\prime }(p)=\frac{2}{3}\text{Re}\;[(U_{0}(p)-2)U_{0}^{\prime }(p)]\;\mathrm{and}\;F_{2,0}^{\prime \prime }(p)=\frac{2}{3}\text{Re}\;[U_{0}^{\prime }{\left(p\right)}^{2}+(U_{0}(p)-2)U_{0}^{\prime \prime }(p)].$$

**Figure 5.**
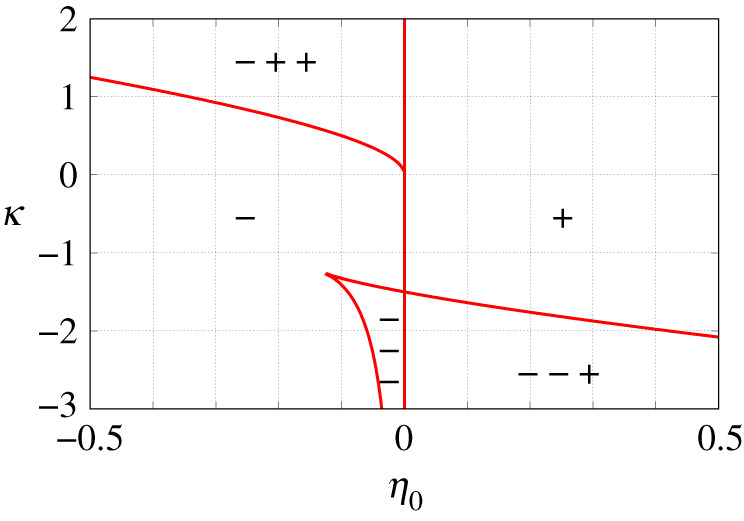
Number of solutions of equation (6.2) with γ=0 and n=2 for different values of the parameters $\eta_{0}$ and κ. The symbols ‘+’ and ‘−’ indicate the number of solutions and their signs in different regions bounded by the solid red curves. (Online version in colour.)

The conclusion of proposition 6.1, in this case, is represented geometrically in figure 5.

### (b) Stability of uniform states

Let $w(x)=p_{0}\in R$ be a constant solution of equation (4.5). It corresponds to the equilibrium $z=U_{\gamma }(p_{0})$ of equation (2.1), and therefore we can perform its stability analysis according to the approach described in §5. Using proposition 5.1, we find that the essential spectrum $\sigma_{\text{ess}}$ consists of two points $\mu_{0}$ and ${\bar{\mu }}_{0}$ where $\mu_{0}=2i\sqrt{p_{0}+i\gamma }$.

To calculate the discrete spectrum $\sigma_{\text{discr}}$, we need to consider equation (5.6). Note that the matrix L(x,λ) of a uniform state does not depend on x. Moreover, simple calculations yield

$${\text{B}}_{\text{1k}}(\lambda )=\delta_{1k}\text{L}(\cdot ,\lambda )$$

and

$${\text{B}}_{\text{jk}}(\lambda )=A\delta_{jk}\text{L}(\cdot ,\lambda )/2\;\text{for}\;j=2,3,$$

where $\delta_{jk}$ is the Kronecker delta. Therefore, equation (5.6) factorizes into three independent equations

6.4 $$\begin{array}{rl} & \;\det({\text{I}}_{2}-{\text{B}}_{\text{11}}(\lambda ))=0, \end{array}$$

6.5 $$\begin{array}{rl} & \;\det({\text{I}}_{2}-{\text{B}}_{\text{22}}(\lambda ))=0, \end{array}$$

6.6 $$\begin{array}{rl} \;\; & \;\det({\text{I}}_{2}-{\text{B}}_{\text{33}}(\lambda ))=0. \end{array}$$

Note that equations (6.5) and (6.6) obviously coincide with each other and therefore determine identical eigenvalues.

**Proposition 6.4.**

*Equation* (6.4) *has two solutions*

$$\lambda_{1,\pm }=\text{Re}\;(\mu_{0}+2\zeta_{0})\pm \sqrt{4|\zeta_{0}{|}^{2}-{\left[\text{Im}\;(\mu_{0}+2\zeta_{0})\right]}^{2}},$$

*while equation* (6.5) *has two solutions*

$$\lambda_{2,\pm }=\text{Re}\;(\mu_{0}+A\zeta_{0})\pm \sqrt{A^{2}|\zeta_{0}{|}^{2}-{\left[\text{Im}\;(\mu_{0}+A\zeta_{0})\right]}^{2}},$$

*where*

$$\zeta_{0}=\frac{\kappa i}{4}D_{n}^{\prime }(a_{0}){\left(1+a_{0}\right)}^{2}\;\text{with}\;a_{0}=U_{\gamma }(p_{0}).$$

**Proof.**

Let us consider equation (6.5). Using the definition of ${\text{B}}_{\text{jk}}(\lambda )$, we rewrite it in the form

$$\det\left[{\text{I}}_{2}-A{\left(\begin{array}{cc} \lambda -\mu_{0} & 0\\ 0 & \lambda -{\bar{\mu }}_{0} \end{array}\right)}^{-1}\left(\begin{array}{cc} \zeta_{0} & \zeta_{0}\\ {\bar{\zeta }}_{0} & {\bar{\zeta }}_{0} \end{array}\right)\right]=0.$$

For every $\lambda \notin \sigma_{\text{ess}}$ (that means $\lambda \neq \mu_{0}$ and $\lambda \neq {\bar{\mu }}_{0}$), the latter equation is equivalent to

$$\begin{array}{rl} & \;\det\left[\left(\begin{array}{cc} \lambda -\mu_{0} & 0\\ 0 & \lambda -{\bar{\mu }}_{0} \end{array}\right)-A\left(\begin{array}{cc} \zeta_{0} & \zeta_{0}\\ {\bar{\zeta }}_{0} & {\bar{\zeta }}_{0} \end{array}\right)\right]\\ & \;\;=(\lambda -\mu_{0}-A\zeta_{0})(\lambda -{\bar{\mu }}_{0}-A{\bar{\zeta }}_{0})-A^{2}|\zeta_{0}{|}^{2}\\ & \;\;=\lambda^{2}-2\lambda \text{Re}\;(\mu_{0}+A\zeta_{0})+|\mu_{0}+A\zeta_{0}{|}^{2}-A^{2}|\zeta_{0}{|}^{2}=0. \end{array}$$

Solving it with respect to λ we obtain the formula for $\lambda_{2,\pm }$. The other equation (6.4) can be considered by analogy.

**Remark 6.5.**

Using (4.4), it is easy to demonstrate that

$$\frac{i}{4}{\left(1+a_{0}\right)}^{2}=-i\sqrt{p_{0}+i\gamma }U_{\gamma }^{\prime }(p_{0})=-\frac{\mu_{0}}{2}U_{\gamma }^{\prime }(p_{0})$$

and

$$\zeta_{0}=-\frac{\mu_{0}}{2}\kappa D_{n}^{\prime }(a_{0})U_{\gamma }^{\prime }(p_{0})=-\frac{\mu_{0}}{2}\kappa {\frac{d}{dp}D_{n}(U_{\gamma }(p))|}_{p=p_{0}}.$$

Let us consider the equation

$$\lambda^{2}-2\lambda \text{Re}\;(\mu_{0}+2\zeta_{0})+|\mu_{0}+2\zeta_{0}{|}^{2}-4|\zeta_{0}{|}^{2}=0$$

that determines $\lambda_{1,\pm }$ and check when it has at least one zero solution. This situation corresponds to a static bifurcation of the uniform state $a_{0}=U_{\gamma }(p_{0})$ and is equivalent to the condition

$$|\mu_{0}+2\zeta_{0}{|}^{2}-4|\zeta_{0}{|}^{2}=0.$$

Inserting here the expression of $\zeta_{0}$ from remark 6.5, we obtain

$$|\mu_{0}{|}^{2}\;(1-2\kappa \text{Re}\;[D_{n}^{\prime }(a_{0})U_{\gamma }^{\prime }(p_{0})])=0.$$

The case $\mu_{0}=0$ corresponds to the degenerate essential spectrum and therefore we discard it. Then the only possibility to satisfy the above equation is to have

$$1-2\kappa \text{Re}\;[D_{n}^{\prime }(a_{0})U_{\gamma }^{\prime }(p_{0})]=1-\kappa F_{n,\gamma }^{\prime }(p_{0})=0.$$

It is easy to verify that the latter condition is equivalent to the situation when the line in the left-hand side of equation (6.2) is tangent to the graph of $F_{n,\gamma }(p)$. In other words, all static bifurcations described by equation (6.4) occur at the fold points of the graph of p versus $\eta_{0}$ with fixed κ.

**Proposition 6.6.**

*In the case* γ=0, *the eigenvalues* $\lambda_{1,\pm }$ *determined by equation* (6.4) *are either both real or complex-conjugate and purely imaginary. Therefore equation* (6.4) *in this case can determine only static bifurcations, i.e. bifurcations characterized by real positive eigenvalues appearing from zero*.

**Proof.**

If $p_{0}>0$, then $\mu_{0}=2i\sqrt{p_{0}}$ and the derivatives $U_{0}^{\prime }(p_{0})$ and $D_{n}^{\prime }(U_{0}(p_{0}))$ are both real. Hence, the value of $\zeta_{0}$ is purely imaginary and therefore

$$\lambda_{1,\pm }=\pm \sqrt{4|\zeta_{0}{|}^{2}-{\left[\text{Im}\;(\mu_{0}+2\zeta_{0})\right]}^{2}}.$$

On the other hand, if $p_{0}<0$, then $\mu_{0}=-2\sqrt{-p_{0}}$ and therefore

$$\lambda_{1,\pm }=\mu_{0}+\text{Re}\;(2\zeta_{0})\pm \sqrt{4|\zeta_{0}{|}^{2}-{\left[\text{Im}\;(2\zeta_{0})\right]}^{2}}=\mu_{0}+\text{Re}\;(2\zeta_{0})\pm |\text{Re}\;(2\zeta_{0})|.$$

This ends the proof.

Propositions 6.1 and 6.6 give a complete description of the uniform states in equation (2.1) with γ=0 and A=0. Indeed, if A=0, then equations (6.5) and (6.6) have no solutions, and therefore the stability of the uniform state is determined by the roots of equation (6.4) only. One can ask what happens to these states if we slightly increase the parameter γ in equation (2.1)? To answer this question, we can use the fact that the parametric representation (6.1) as well as the explicit formula of eigenvalues $\lambda_{1,\pm }$, see proposition 6.4, in general smoothly depend on γ. Therefore, if $\max(\text{Re}\;\lambda_{1,-},\text{Re}\;\lambda_{1,+})<0$ or $\max(\text{Re}\;\lambda_{1,-},\text{Re}\;\lambda_{1,+})>0$, then the same inequality will remain true for small enough values of γ. However, a similar argument does not work in the case of complex-conjugate and purely imaginary eigenvalues $\lambda_{1,\pm }$. From proposition 6.6, it follows that such a situation occurs if $p_{0}>0$ and $4|\zeta_{0}{|}^{2}-{\left[\text{Im}\;(\mu_{0}+2\zeta_{0})\right]}^{2}<0$. We consider this case in more detail below.

**Remark 6.7.**

If γ=0 and $p_{0}>0$, then $\mu_{0}=2i\sqrt{p_{0}}$ and $\zeta_{0}$ (see remark 6.5) are purely imaginary, therefore

$$\begin{array}{rl} 4|\zeta_{0}{|}^{2}-{\left[\text{Im}\;(\mu_{0}+2\zeta_{0})\right]}^{2} & \;=\mu_{0}^{2}+4\mu_{0}\zeta_{0}\\ & \;=\mu_{0}^{2}\left(1-2\kappa {\frac{d}{dp}D_{n}(U_{0}(p))|}_{p=p_{0}}\right)=-4p_{0}(1-\kappa F_{n,0}^{\prime }(p_{0})). \end{array}$$

Hence, in this case, the inequality $4|\zeta_{0}{|}^{2}-{\left[\text{Im}\;(\mu_{0}+2\zeta_{0})\right]}^{2}<0$ is equivalent to $1-\kappa F_{n,0}^{\prime }(p_{0})>0$.

**Proposition 6.8.**

*Let* $p_{0}>0$ *and* $1-\kappa F_{n,0}^{\prime }(p_{0})>0$. *Then for sufficiently small* γ>0, *there exists a uniform state* $z=U_{\gamma }(p_{0})$ *of equation* (2.1) *with* $\eta_{0}=p_{0}-\kappa F_{n,\gamma }(p_{0})$. *Moreover, the eigenvalues* $\lambda_{1,\pm }$ *are complex conjugate and satisfy*

Re λ1,±<0,if 2>κ(Fn,0′(p0)+2p0Fn,0″(p0))

*and*

Re λ1,±>0,if 2<κ(Fn,0′(p0)+2p0Fn,0″(p0)).

**Figure 6.**
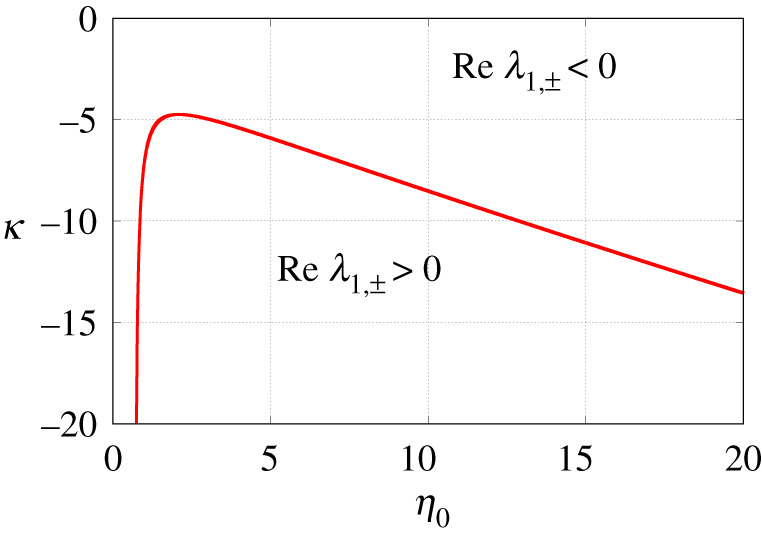
Graphical representation of the assertion of proposition 6.8 for n=2. The solid red curve has a vertical asymptote $\eta_{0}=2/3$ at the left end. (Online version in colour.)

*See* *figure 6* *for a graphical representation of this proposition*.

**Proof.**

The existence of the uniform state $z=U_{\gamma }(p_{0})$ is obvious and we need to consider only the corresponding expression

6.7 $$\lambda_{1,\pm }=\text{Re}\;(\mu_{0}+2\zeta_{0})\pm \sqrt{4|\zeta_{0}{|}^{2}-{\left[\text{Im}\;(\mu_{0}+2\zeta_{0})\right]}^{2}},$$

taking into account that $\mu_{0}$ and $\zeta_{0}$ depend on γ. Due to the assumptions $p_{0}>0$ and $1-\kappa F_{n,0}^{\prime }(p_{0})>0$ we can be sure that the square root in equation (6.7) remains purely imaginary for sufficiently small γ, therefore

$$\text{Re}\;\lambda_{1,\pm }=\text{Re}\;(\mu_{0}+2\zeta_{0}).$$

On the other hand, using the formula $\mu_{0}=2i\sqrt{p_{0}+i\gamma }$ and remark 6.5, we obtain

$${\frac{d\mu_{0}}{d\gamma }|}_{\gamma =0}=-\frac{1}{\sqrt{p_{0}}}\;\mathrm{and}\;{\frac{d\zeta_{0}}{d\gamma }|}_{\gamma =0}=\frac{\kappa }{2\sqrt{p_{0}}}{\frac{d}{dp}D_{n}(U_{0}(p))|}_{p=p_{0}}+\kappa \sqrt{p_{0}}{\frac{d^{2}}{dp^{2}}D_{n}(U_{0}(p))|}_{p=p_{0}}.$$

This implies that

$$\text{Re}\;\lambda_{1,\pm }=-\frac{2-\kappa (F_{n,0}^{\prime }(p_{0})+2p_{0}F_{n,0}^{\prime \prime }(p_{0}))}{2\sqrt{p_{0}}}\gamma +O(\gamma^{2})$$

for γ→0.

Proposition 6.8 has an important consequence for the stability of the uniform states of equation (2.1). Although for γ=0 such states can undergo only static bifurcations (see proposition 6.6), for γ>0 they can lose stability through a Hopf bifurcation too. In particular, for small γ, a Hopf bifurcation occurs in the vicinity of the curve described parametrically by the equations

$$\kappa =2{\left(F_{n,0}^{\prime }(p)+2pF_{n,0}^{\prime \prime }(p)\right)}^{-1}\;\mathrm{and}\;\eta_{0}=p-\kappa F_{n,0}(p)$$

with p>0 that satisfy the inequality $1-\kappa F_{n,0}^{\prime }(p)>0$. An example of such a curve for n=2 is shown in figure 6.

## 7. Spatially non-uniform states

In the previous section, we considered the constant equilibria of equation (2.1), which correspond to uniform rest states and uniform spiking states of coupled theta neurons (1.1). However, these are in general not the only possible solutions of equation (2.1). Below we show that for a cosine coupling (1.2) with A≠0 equation (2.1) also has a variety of non-constant equilibria representing more complex patterns in system (1.1). We focus on the case γ=0, n=2 and test three distinct values κ=1, −1 and −2 corresponding to three qualitatively different scenarios in figure 5. For each of these cases, we consider three distinct values of A, namely 0, 3 and −5.

**Figure 7.**
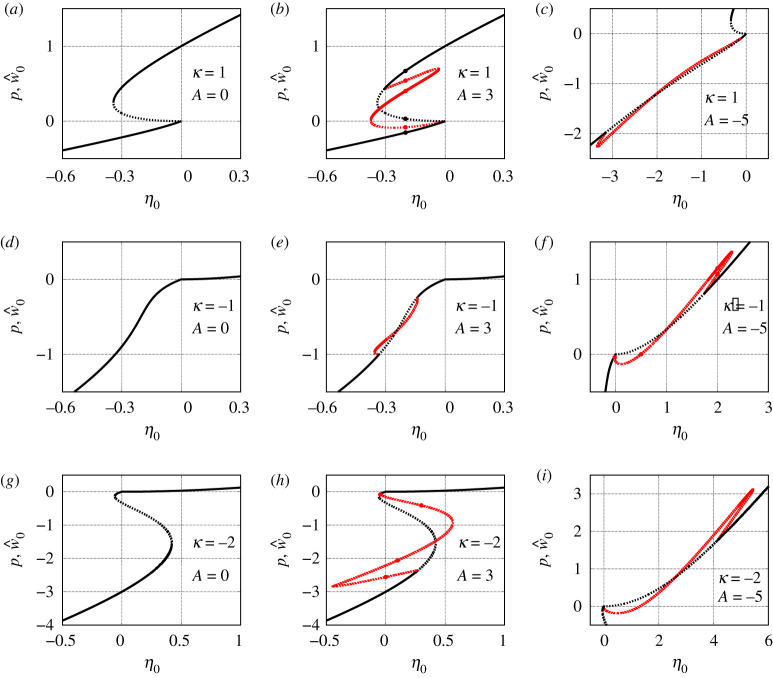
Solution branches of equation (6.2) (for spatially uniform states) or of (4.7) and (4.8) (for non-uniform states) with γ=0 and n=2 for different pairs of the parameters κ and A. Solid and dotted curves show stable and unstable solutions. Black curves correspond to uniform states, while red curves correspond to spatially modulated states and bump states. The dots in panels (*b*), (*f*) and (*h*) show points corresponding to the solutions shown in figures 8, 9, 12 and 13. In panel (*c*), the red curve has two tiny stable parts close to the fold point on the lower branch and close to the right end of the curve. Panel (*b*) shows a situation analogous to that shown in fig. 9 in [21].

In figure 7, for all nine combinations of κ and A values, we plot either p (for spatially uniform states) or ${\hat{w}}_{0}$ (for non-uniform states) as a function of $\eta_{0}$. The branch of uniform states is always independent of A, but the stability of different states on it can change for different A. If A≠0, then using proposition 6.4, we typically find two bifurcation points determined by the condition $\lambda_{2,\pm }=0$. Then, using the self-consistency equations (4.7)–(4.8) we can compute a branch of non-constant equilibria of equation (2.1) that connects these two bifurcation points. Every solution $\left({\hat{w}}_{0},{\hat{w}}_{1}\right)$ of equations (4.7)–(4.8) with ${\hat{w}}_{1}\neq 0$ corresponds to a non-constant even solution (4.6) of equation (4.5) and hence to a spatially modulated equilibrium $a(x)=U_{\gamma }(w(x))$ of equation (2.1). The stability of this equilibrium can be analysed using the results of §5. More precisely, the essential spectrum $\sigma_{\text{ess}}$ of the operator on the right-hand side of the linearized equation (5.1) is determined by proposition 5.1, while the discrete spectrum $\sigma_{\text{discr}}$ of this operator can be calculated by numerically solving the characteristic equation (5.6). Note that since w(x) is an even function, the matrix L(x,λ) in the definition of B(λ) is an even function of x too. Therefore, ${\text{B}}_{\text{jk}}(\lambda )=0$ for j=1,2 and k=3 as well as for j=3 and k=1,2. Consequently, equation (5.6) factorizes into two independent equations

7.1 $$\det\left[{\text{I}}_{4}-\left(\begin{array}{cc} {\text{B}}_{\text{11}}(\lambda ) & {\text{B}}_{\text{12}}(\lambda )\\ {\text{B}}_{\text{21}}(\lambda ) & {\text{B}}_{\text{22}}(\lambda ) \end{array}\right)\right]=0$$

and

7.2 $$\det[{\text{I}}_{2}-{\text{B}}_{\text{33}}(\lambda )]=0.$$

It is easy to verify that, by construction, equation (7.1) determines eigenvalues corresponding to perturbations that do not break the $Z_{2}$-symmetry of the considered equilibrium, while equation (7.2) determines eigenvalues corresponding to symmetry-breaking perturbations. It turns out that all instabilities shown in figure 7 are due to equation (7.1) only. On the other hand, for every non-constant even equilibrium a(x) equation (7.2) yields a zero eigenvalue that is related to the translation invariance of equation (2.1).

**Figure 8.**
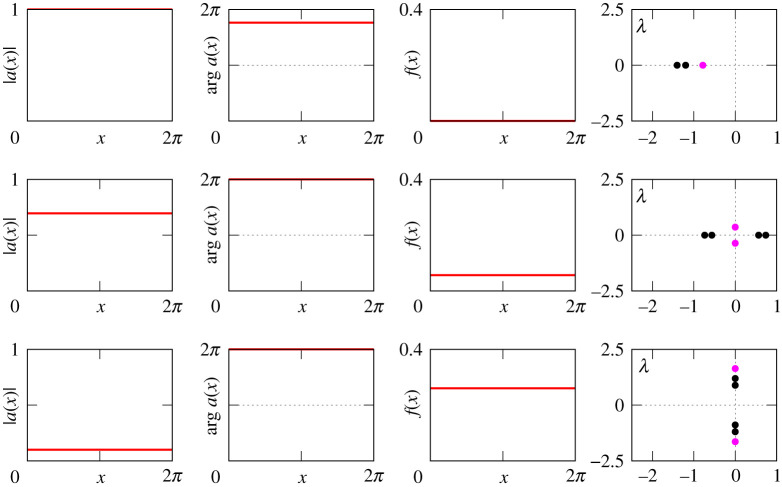
The equilibria a(x) of equation (2.1), the firing rates f(x) and the spectra of the corresponding linearized operator for the three black points in figure 7*b*. The purple and the black dots in the far right panels show essential and discrete spectra, respectively. The top panels correspond to a stable ‘all-off’ state while the bottom panels correspond to a neutrally stable ‘all-on’ state.

**Figure 9.**
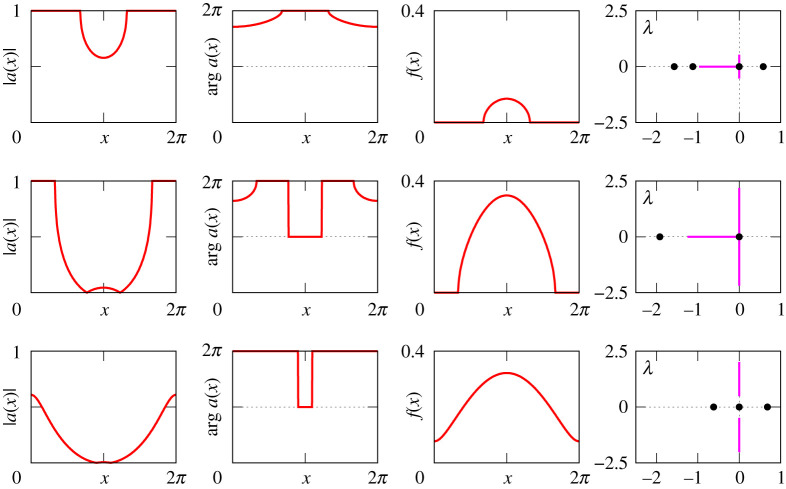
The equilibria a(x) of equation (2.1), the firing rates f(x) and the spectra of the corresponding linearized operator for the three red points in figure 7*b*. The purple lines and the black dots in the far right panels show essential and discrete spectra, respectively. The top panel shows an unstable bump solution, the middle shows a neutrally stable bump, while the bottom shows an unstable spatially modulated state. The solution in the middle panel is analogous to that shown in fig. 2 of [20].

**Figure 10.**
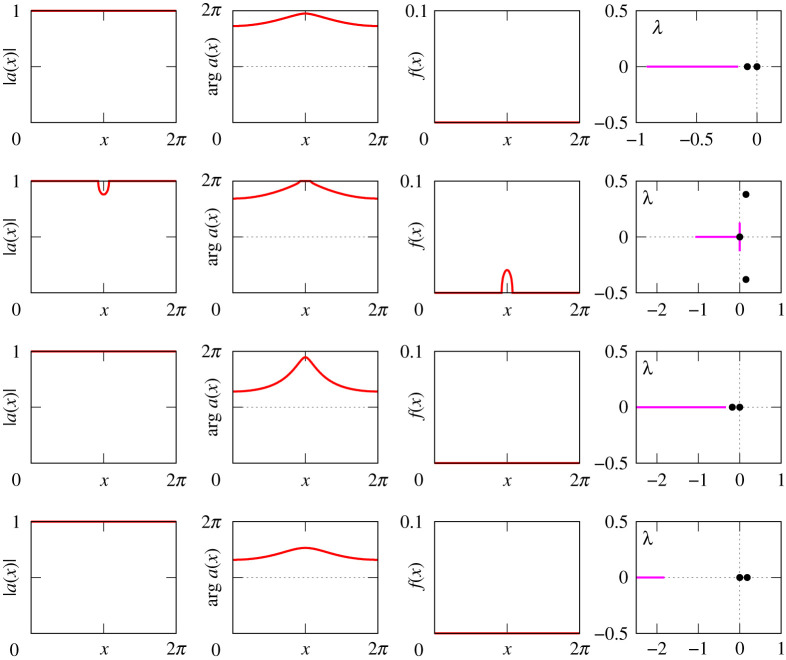
The equilibria a(x) of equation (2.1), the firing rates f(x) and the spectra of the corresponding linearized operator for several points in figure 7*c*. Parameters: $\eta_{0}=-0.14$, −0.2, −3.32 and −3.25 (upper branch).

**Figure 11.**
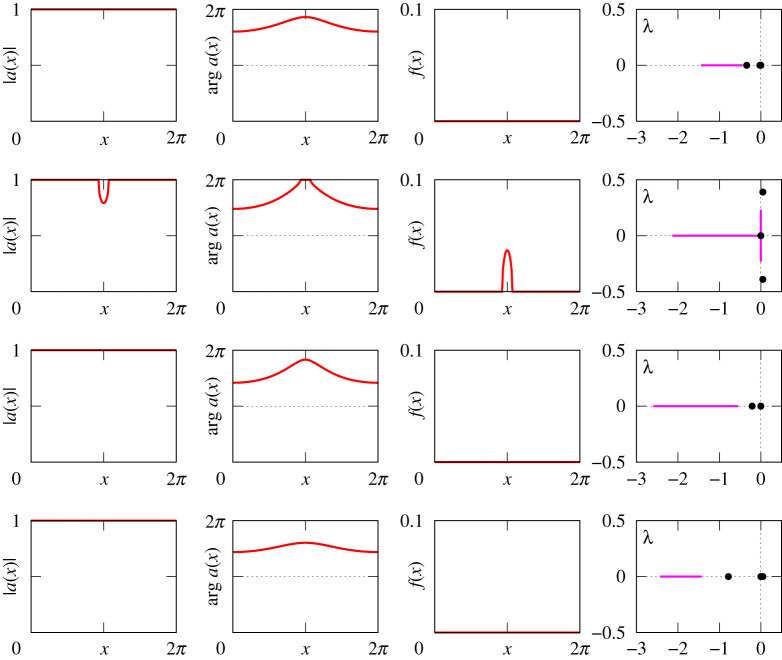
The equilibria a(x) of equation (2.1), the firing rates f(x) and the spectra of the corresponding linearized operator for several points in figure 7*e*. Parameters: $\eta_{0}=-0.14$, −0.2, −0.33 and −0.35 (lower branch).

**Figure 12.**
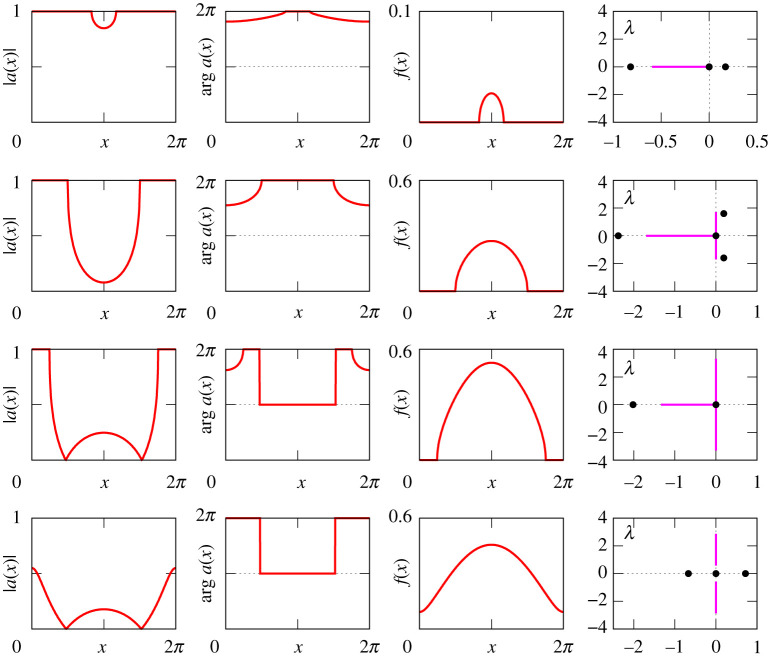
The equilibria a(x) of equation (2.1), the firing rates f(x) and the spectra of the corresponding linearized operator for the four red points in figure 7*f*. The results here are similar to those shown in figure 9. This is not unexpected, since for κ=−1 and A<−1, the effective coupling is of Mexican hat type with a positive value of κ.

**Figure 13.**
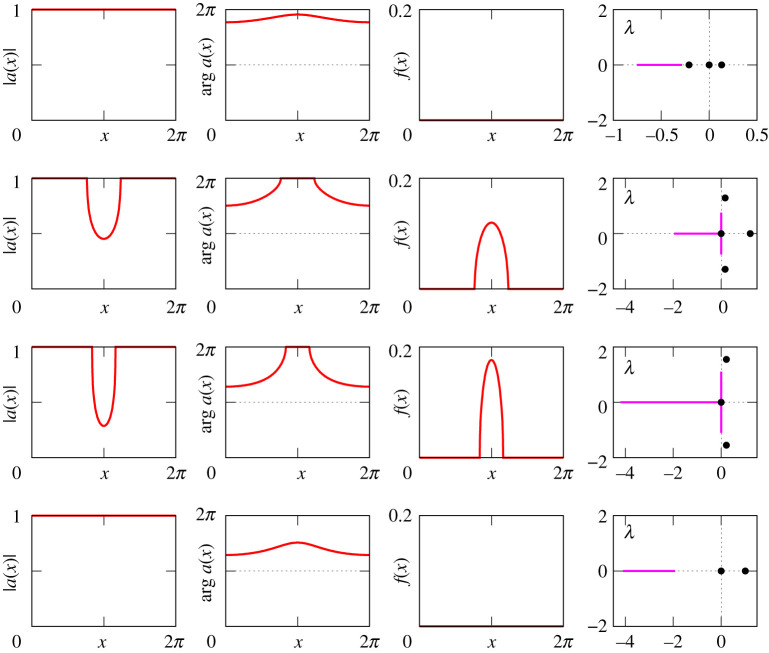
The equilibria a(x) of equation (2.1), the firing rates f(x) and the spectra of the corresponding linearized operator for the four red points in figure 7*h*.

Figures 8–13 provide additional information about the solution branches in figure 7. Each row of these figures shows an equilibrium a(x) of equation (2.1), the corresponding firing rate f(x) calculated by (2.2), and the linear stability spectrum of a(x). The sampled equilibria are chosen so that they illustrate all qualitatively different spatial profiles a(x) and their linear stability properties. Note that we do not show any equilibria for figure 7*i*, because they look very similar to those shown in figure 12.

Our results reveal several parameter combinations suitable for the observation of stable bump states. In particular, such states can be found for (κ,A)=(1,3), (κ,A)=(−1,−5) and (κ,A)=(−2,−5). Although in all these cases, the bump states have similar spatial profiles, their stability diagrams for positive and negative κ are a bit different. More precisely, for κ=1, the $\eta_{0}$-interval of stable bumps is bounded by two fold points, while for κ=−1 (as well as for κ=−2) the stability interval of bumps is bounded by a fold point at the right end and by a Hopf bifurcation at the left end.

Apart from the bump states, equation (2.1) has only one more type of stable spatially non-uniform equilibria. These are modulated rest states shown in the first and third rows of both figures 10 and 11. Even though none of the neurons are firing here, their state is not spatially uniform.

Finally, we emphasize that our consideration reveals also a wide parameter range, see figure 7*c*,*e*,*f*,*i*, where all equilibria of equation (2.1) are unstable. In this case, any simple collective dynamics of neurons is not possible, therefore more complex non-stationary patterns can emerge in system (1.1), see figure 3.

## 8. Discussion

In summary, we analysed the dynamics of a ring network of theta neurons, homogeneously coupled with a cosine kernel. We took the continuum limit that allows us to describe the network’s asymptotic dynamics by a complex-valued integrodifferential equation (2.1). We showed how to determine the existence and stability of stationary solutions, both spatially uniform and non-uniform. We have given a number of examples demonstrating the validity of our approach, mostly concentrating on the case of identical neurons. These results put on a rigorous basis some of the numerical observations in [20,21].

We now discuss how our results relate to those of others. Byrne *et al.* [17] considered either one or two populations of theta neurons with synaptic coupling using reversal potentials and characteristic timescales, and exponentially decaying coupling kernels. They considered domains with either periodic or Neumann boundary conditions and looked for Turing bifurcations of the spatially uniform state. They also performed numerical bifurcation analysis of time-dependent solutions such as wavetrains and travelling fronts. Esnaola-Acebes *et al.* [14] also considered two ring networks of excitatory and inhibitory QIF neurons with even coupling functions involving more than just the first cosine term. They chose parameters so that there was a single spatially uniform state and performed a Turing bifurcation analysis of this state. This analysis was used to explain the decaying oscillatory transient spatial modes seen when the spatially uniform state was perturbed. They also numerically followed a bump solution and found a scenario of the form shown in figure 7*e*.

Byrne *et al.* [16] considered a network of QIF neurons on a periodic domain, with synaptic dynamics and an inverted Mexican hat connectivity. They also included gap junctional coupling. By analysing the continuum equations they found Turing–Hopf bifurcations of the spatially uniform state that result in the appearance of travelling and standing waves. Schmidt & Avitabile [15] studied ring networks of QIF neurons with Mexican hat connectivity and also determined the conditions for a Turing bifurcation.

Most of these works presented largely numerical studies of the fully nonlinear and often time-dependent solutions that such networks can support whereas we have concentrated on stationary solutions, exploiting the form of the coupling function to explicitly calculate these and analytically determine their stability.

Our approach could easily be generalized to other forms of coupling such as synaptic coupling using reversal potentials [17] or gap junctional coupling [16,35], and different coupling kernels, as long as the form of the equations allows the use of the Ott/Antonsen ansatz.

## Data Availability

The codes used for producing the data for figures are available in electronic supplementary material [36].

## Appendix A

Proposition A.1. *For every* c∈R *and* γ≥0, *there exists exactly one square root value* $\xi =\sqrt{c+i\gamma }$ *in the quadrant* Re ξ≥0, Im ξ≥0, *which is given by*

$$\xi =\frac{1}{\sqrt{2}}\left(\sqrt{\sqrt{c^{2}+\gamma^{2}}+c}+i\sqrt{\sqrt{c^{2}+\gamma^{2}}-c}\right).$$

Proof. A complex number c+iγ can be written in the polar form

$$c+i\gamma =\sqrt{c^{2}+\gamma^{2}}\;e^{i\phi }$$

where

$$\cos\phi =\frac{c}{\sqrt{c^{2}+\gamma^{2}}}\;\mathrm{and}\;\sin\phi =\frac{\gamma }{\sqrt{c^{2}+\gamma^{2}}}.$$

Since γ≥0, we have ϕ∈[0,π]. Therefore,

A\,1 $$\xi =\sqrt[{4}]{c^{2}+\gamma^{2}}\;e^{i\phi /2}$$

is the value of the square root $\sqrt{c+i\gamma }$ that lies in the quadrant Re ξ≥0, Im ξ≥0 (the other value of the square root has the opposite sign and therefore satisfies Re ξ≤0 and Im ξ≤0). Using standard trigonometric identities and the fact that ϕ∈[0,π], we find

$$\begin{array}{rl} & \cos\frac{\phi }{2}=\sqrt{\frac{1+\cos\phi }{2}}=\sqrt{\frac{1}{2}\left(1+\frac{c}{\sqrt{c^{2}+\gamma^{2}}}\right)}\\ \mathrm{and}\;\; & \sin\frac{\phi }{2}=\sqrt{\frac{1+\cos\phi }{2}}=\sqrt{\frac{1}{2}\left(1-\frac{c}{\sqrt{c^{2}+\gamma^{2}}}\right)}. \end{array}$$

Inserting these expressions into formula (A 1), we obtain the assertion of the proposition.
